# Detecting within-host interactions from genotype combination prevalence data

**DOI:** 10.1016/j.epidem.2019.100349

**Published:** 2019-12

**Authors:** Samuel Alizon, Carmen Lía Murall, Emma Saulnier, Mircea T. Sofonea

**Affiliations:** MIVEGEC, CNRS, IRD, Université de Montpellier, France

**Keywords:** Multiple infections, MOI, Superspreaders, Inference, ABC, Competition

## Abstract

Parasite genetic diversity can provide information on disease transmission dynamics but most mathematical and statistical frameworks ignore the exact combinations of genotypes in infections. We introduce and validate a new method that combines explicit epidemiological modelling of coinfections and regression-Approximate Bayesian Computing (ABC) to detect within-host interactions. Using a susceptible-infected-susceptible (SIS) model, we show that, if sufficiently strong, within-host parasite interactions can be detected from epidemiological data. We also show that, in this simple setting, this detection is robust even in the face of some level of host heterogeneity in behaviour. These simulations results offer promising applications to analyse large datasets of multiple infection prevalence data, such as those collected for genital infections by Human Papillomaviruses (HPVs).

## 1 Introduction

Hosts are known to often be simultaneously infected by multiple genotypes of the same parasite species or even by multiple parasite species. Here, we use the generic definitions for parasite, which refers to both micro- and macroparasites, and genotype, which refers to any genetic variant. Over the last decades, the gap between our ability to detect this parasite within-host diversity and its use in epidemiological inference model has widened. Indeed, the affordability and applicability of sequencing technologies have progressed much more rapidly than the ability for epidemiological models to account for within-host microbial diversity. Here, we introduce and validate an approach to detect within-host interaction from equilibrium prevalence data even in the presence of another source of heterogeneity, namely differences in host behaviour. This method relies on the exact combination of parasite genotypes in each host, which we from here on refer to as the ‘genotype combination’. We use the spread of genital infections by different types of human papillomaviruses (HPVs) as an example because these are known to cause many multiple infections and are closely monitored because of their potential carcinogenicity (Thomas et al., 2000, Rousseau et al., 2001, Chaturvedi et al., 2011). However, this method is applicable to many other host-parasite systems with high prevalence of multiple infections and dense sampling.

### 1.1 Binary or rank models

Most epidemiological models with parasite genotype coexistence within hosts only allow for up to two genotypes per host and do not allow for cotransmission, although there are exceptions for both (May and Nowak, 1995, Lion, 2013, Alizon, 2013, Sofonea et al., 2015). These ‘binary’ models have been instrumental in epidemiology but are by definition inappropriate as soon as parasite diversity exceeds two genotypes.

Conversely, studies on macro-parasites have long been incorporating the multiplicity of infection in their models (Anderson and May, 1978). They showed that the distribution of the number of macro-parasites per host can provide information regarding the contact structure within the host population. In absence of heterogeneity of any kind, one would expect to detect Poisson distributions. Interestingly, in many populations, the number of macro-parasites per host is best explained by a negative-binomial distribution, which is often interpreted as evidence for some sort of host population structure (Shaw and Dobson, 1995, Wilber et al., 2017). This aggregation pattern then shapes the functional response between parasitism and host death rate in ways that can critically affect population dynamics (Anderson and May, 1978).

**Fig. 1:**
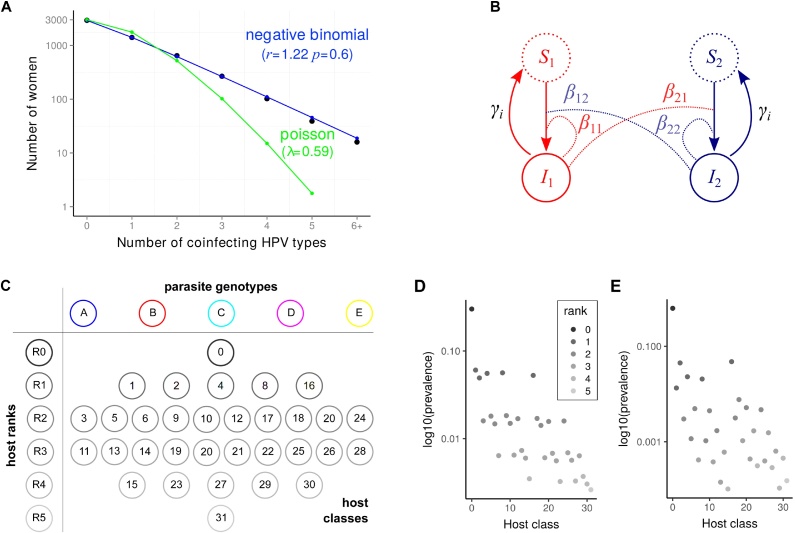
The coinfection epidemiological setting. (A) Empirical rank distribution for HPV infections, (B) flow diagram showing the population structure with ‘normal-spreader’ (1 in red) and ‘super-spreader’ hosts (2 in dark blue), (C) host class prevalences for *n* = 5 genotypes, (D) combination prevalences for a scenario with weak (*k* ≈ 0.02) and (E) with strong interaction (*k* = ≈ −0.41). In A, black dots show data from 5412 sexually active women in the Costa Rica Vaccine Trial reported by Chaturvedi et al. (2011) and lines show maximum likelihood fits performed using the bbmle package in R (Bolker, 2008). In B, the *β* and *γ* indicate transmission and recovery rates. In C, each circle indicates a prevalence (per genotype, per rank or per combination) that can be used as a summary statistics. Numbers in the combination correspond to a binary code indicating the nature of the genotypes present. In D and E, the shading indicates the infection rank (or number of coinfecting genotypes) and the class is a binary code indicating the genotypes present. We assume that genotypes B and E are less competitive than genotypes A, C and D.

For microparasites, similar studies have been developed, where the number of macroparasites per host corresponds to the number of genotypes detected in a host, which we refer to as the infection ‘rank’. For example, Chaturvedi et al. (2011) showed that a Poisson distribution can be rejected for HPV genital infections suggesting that there is an excess of coinfections compared to what would be expected in a standard Susceptible-Infected (SI) model. Additional analyses of ours show that a negative binomial distribution helps capture the tail of this distribution (Fig. 1A). This is consistent with the fact that the ‘number of lifetime partners’ was the cofactor the most strongly associated with being infected by multiple HPV types instead of a single HPV type in the study by Chaturvedi et al.

Fenton et al. (2014) compared several techniques using a dataset involving two species for which real within-host interactions were known from laboratory experiments. They concluded that correlation techniques performed worse and that the best method required time series and not just cross-sectional data (see Shrestha et al., 2011 on how to infer interaction parameters from time series using particle filtering techniques). In general, longitudinal data allows for more detailed epidemiological inference than equilibrium data (Rohani and King, 2010). However, the restricted number of strains they used also potentially limited the power of their conclusion (3 ranks and 2 total prevalences versus 4 combinations).

### 1.2 Parasite combination prevalences

Intuitively, there should be more information in the prevalence of each combination of genotypes than in the rank prevalence. With 5 circulating genotypes, there are only 6 possible ranks but 32 genotype combinations (Fig. 1C). Earlier studies have already thought about using this data to compensate for the lack of longitudinal data. In particular, Vaumourin et al. (2014) considered systems with a larger number of genotypes using a variety of existing techniques (generalised chi-square, network models and multinomial GLM approaches) and developed a new association screening approach that has the advantage to identify and sort combinations based on their deviation from the expectation (see Section 2). Essentially, their methods consists in testing whether the observed genotype combination prevalence distribution significantly differs from the ‘neutral’ distribution in which parasites do not interact in their host (also referred to as ‘*H*_0_’). This neutral distribution is built from the total prevalence of each genotype assuming a multinomial distribution. As the Poisson distribution used by (Chaturvedi et al., 2011), it implicitly assumes an SI model with co-transmission.

One of the limitations of not having an explicit epidemiological model is that any type of heterogeneity into the system may lead to a deviation from *H*_0_. In particular, infected hosts may differ in their phenotypes for reasons other than the nature of the genotype(s) infecting them. Detecting an effect of interactions between genotypes on equilibrium prevalences therefore requires ruling out other important sources of host heterogeneity.

### 1.3 Inference using explicit modelling

Our goal in this study is twofold. First, we want to assess the additional information that can be obtained from genotype combination data. Second, we want to control for another source of host heterogeneity, namely the fact that some hosts may act as ‘super-spreaders’ (Lloyd-Smith et al., 2005). As mentioned above (Chaturvedi et al., 2011), these hosts should be more exposed to the infection and therefore have higher infection ranks independently of any features of the parasites themselves. Our hypothesis is that using a mathematical model that captures the epidemiological dynamics of *n* parasite genotypes (or species) in their 2^*n*^ host classes allows us to address both our goals simultaneously.

Although our approach can be applied to many systems, we focus on modelling scenarios similar to genital infections caused by different types of human papillomaviruses (HPVs) for reasons that are detailed in Supplementary Information. We consider that the population contains *n* = 5 different genotypes, which we track individually. There are therefore 32 host classes in the population.

Our goal is to estimate the magnitude of the interaction term between some genotypes. To this end, we adopt a mechanistic approach and simulate epidemiological coinfection dynamics with 5 genotypes. This is made possible by a recent analytical framework that can handle an arbitrary number of genotypes (Sofonea et al., 2015). In order to assess the ability to infer interactions from the observed coinfection classes, we use a regression-based Approximate Bayesian Computing (ABC) approach (Csilléry et al., 2012, Saulnier et al., 2017). We show that our method performs well on simulated data and can distinguish overall genotype interactions even in the presence of host behavioural heterogeneity.

## 2 Methods

### 2.1 The epidemiological model

The model is based on the deterministic ODE-based framework introduced by Sofonea et al., 2015Sofonea et al. (2015) that allows for an arbitrary number of parasite genotypes to circulate in a host population without assuming any particular infection pattern (see Sofonea et al., 2017Sofonea et al. (2017) for the importance of this relaxation). Furthermore, the framework enables cotransmission in the sense that infected hosts can simultaneously transmit any subset of genotypes they are infected with.

#### 2.1.1 Multiple infections

Let us consider that hosts can be potentially infected by any combination of *n* parasite genotypes and sort them in classes according to the genotypes present (we use a binary code to map the presence/absence of the genotypes the hosts class labels). For computational reasons, we assume in the simulations that *n* ≤ 5, as the number of classes increases geometrically with the number of genotypes.

Epidemiological dynamics follow a classical susceptible-infected-susceptible (SIS) framework, where upon contact with an infected host, a ‘recipient’ host can acquire any subset of the genotypes carried by this ‘donor’ host (cotransmission). In terms of recovery, we assume that genotypes can only be cleared one at a time and independently, unless there are within-host interactions. In the case of HPVs, the average infection duration for acute infections is estimated to be in the order of magnitude of a year (Insinga et al., 2007, Trottier et al., 2008). Given that we focus on HPV infections in young adults, we neglect infection-induced mortality.

Mathematically, the dynamics can be captured in a compact form using the master equation (Sofonea et al., 2015):

(1) $$d\text{y}/dt=\beta \Phi (\text{y}⊗\text{y})-\beta (\Psi \text{y})⊙\text{y}+\left(\Xi -\Theta \right)\text{y}$$

where **y** is the vector of densities of the 2^*n*^ host classes, ⊙ denotes the Hadamard element-wise matrix product, ⊗ the Kronecker (outer) product, **Φ** is the infection input flow matrix, **Ψ** is the infection output flow matrix, **Ξ** is the recovery input flow matrix and **Θ** is the recovery output flow matrix and *β* is the (constant) probability of transmission per contact that scales all infection processes. Note that all the heterogeneity in infections comes from the recovery matrix. Each genotype has its own recovery rate (*d*_*i*_), which can be impacted by the presence of other genotypes in the host. Further details about this equation can be found in Supplementary Information and in Sofonea et al. (2015).

Equation (1) allows us to track all the flows going in and out of host compartments through time. For simplicity, we neglect host demography (births and deaths) and assume that the host population size is constant. HPV infected hosts do not always sero-convert and natural immunity is lower than vaccine-induced immunity (Beachler et al., 2016) so we neglect immunisation in the model. We also neglect vaccination, but it could be readily considered by either assuming that one of the host types is vaccinated or by doubling the number of host types.

#### 2.1.2 Population structure

The model was enhanced by splitting the host population into two sub-populations that differ in their contact rates (‘super-spreader’ versus ‘normal-spreader’ hosts) as shown in Fig. 1B (Keeling and Rohani, 2008). With 5 genotypes, we therefore have 64 host classes instead of 32 in a homogeneous population. Contacts between the two sub-populations follow a classical pattern based on the assortment (*a*) within host types, the proportion of each host type (*p*_1_ = *p* and *p*_2_ = 1 − *p*) and their activity rates (equal to *c*_1_ = 1 and *c*_2_ = *h*, with *h* ≥ 1). Overall, the contact rate between a ‘recipient’ individual from sub-population *j* and a ‘donor’ individual from sub-population *i* is

(2) $$c_{ji}=(1-a)\frac{c_{i}\;c_{j}}{p+(1-p)\;h}+\delta_{ij}\;a\;c_{i}$$

where *δ*_*ij*_ is the Kronecker delta and *h* is the difference in activity between the two host types. The two terms of the right hand side indicate that contacts between *i* and *j* can be due to random contacts (the first term) or driven by assortment if *i* = *j* (the second term).

This population structure implies that we have two vectors of host classes (**y_1_** and **y_2_**). If we denote the combined vector ${\text{y}}_{•}=\left({\text{y}}_{1},{\text{y}}_{2}\right)$, the master equation can be written similarly to (1) by updating the matrices in the following way:

$$\begin{array}{cc} {\text{A}}_{•} & =\mathrm{diag}\left(\text{A},\text{A}\right)\;\mathrm{for}\;\text{A}\equiv \Theta ,\Xi ,\\ \Psi_{•} & =\left[\begin{array}{cc} c_{11} & c_{12}\\ c_{21} & c_{22} \end{array}\right]⊗\Psi \;\mathrm{and}\\ \Phi_{•} & =\left[\begin{array}{cc} \left(11^{T}⊗\left(c_{11},c_{12}\right)⊗1^{T}\right)⊙\Phi^{\prime } & 0\\ 0 & \left(11^{T}⊗\left(c_{21},c_{22}\right)⊗1^{T}\right)⊙\Phi^{\prime } \end{array}\right], \end{array}$$

where **1** denotes the 2^*n*^-dimensional column vector with unit elements, and **Φ**′ is obtained by repeating each 2^*n*^ × 2^*n*^ block **Φ**^[*i*]^ of the original 2^*n*^ × 2^2*n*^ matrix $\Phi ={\left(\Phi^{\left[i\right]}\right)}_{i=1,\ldots ,2^{n}}$ as $\Phi^{\prime }={\left(\Phi^{\left[i\right]},\Phi^{\left[i\right]}\right)}_{i=1,\ldots ,2^{n}}$.

#### 2.1.3 Model simulations

The model equations were implemented in R and 50,001 simulations were run, each with different parameter combinations to use as a training dataset for the ABC. The script can be found in Supplementary Information along with the raw data on simulated prevalences.

The equilibrium prevalences from the deterministic model were used to generate datasets in finite populations of 1000, 5000 and 10,000 hosts assuming a multinomial distribution, where the probability to draw a host with a given genotype combination was equal to this combination's prevalence.

#### 2.1.4 Genotype interactions

In our epidemiological model, we neglected the temporal dynamics of the within-host processes and summarised them into constant parameters. This absence of within-host component means that we are unable to detect a specific interaction (e.g. discriminating between cross-immunity and resource competition) and only analyse the overall effect of all within-host interactions between genotypes.

We assumed that genotype transmission rates were identical and unaffected by the presence of other genotypes. This was motivated by the very high transmission probability of HPV per contact (Winer et al., 2006). We therefore assumed that interactions between genotypes occurred through the recovery rates.

Even with 5 genotypes, this could mean introducing 20 unknown interaction parameters (e.g. how the presence of genotype 1 affect the clearance rate of genotype 2). To reduce this complexity, we sorted genotypes into two groups with different competitive abilities. Whenever a genotype from to the least competitive group coinfects a host with a genotype from to the most competitive group, its individual recovery rate (*d*_*i*_) is multiplied by a factor 1 + *k*, with *k* ∈ [−0.5, 0.5]. Recovery rates are unaffected (*k* = 0) if all the genotypes in the host belong to the least competitive group. Genotypes from the most competitive group are always unaffected by the presence of other genotypes. If *k* > 0, host classes containing genotypes from the least competitive group to be under-represented. The reverse is true if *k* < 0. We assumed that one of the groups contained 3 genotypes and the other 2. Biologically speaking, the two groups could correspond to High-Risk (HR) and Low-Risk (LR) HPV types. Another possibility would be to compare HPV16 and HPV18, which together account for the vast majority of HPV-driven cancers, to the other HPV types.

### 2.2 Inference from distributions

In order to compare our framework to existing methods, we used the techniques implemented by Vaumourin et al. (2014) in R. Three of these, which are the less computationally intensive, are briefly described here but readers interested in more details should refer to the original publication. For each of these techniques, we analysed a dataset with two host types (normal-spreaders and super-spreaders) and a dataset with a unique host type. Our hypothesis is that these methods should not be able to distinguish between the heterogeneity caused by the genotype within-host interactions and that caused by host behaviour.

#### 2.2.1 Association screening

This approach involves simulating datasets of occurrence count of each combination of genotype based on the genotype prevalences (Vaumourin et al., 2014). From these simulations, a 95% confidence envelope is calculated for each combination, thus allowing to detect deviation from the expected distribution in the dataset (also referred to as *H*_0_).

#### 2.2.2 Multinomial GLM

This model consists in calculating the deviance from a statistical distribution obtained with a Generalised Linear Model and a multinomial family. Practically, the multinomial logistic regression model was performed using the *vglm* function from the VGAM package in R (Yee, 2015).

#### 2.2.3 Generalised chi-square

This test does not involve any simulations and is based on the expected chi-square distribution of the prevalence of each combination of genotype given the total prevalence of each genotype. Note that combinations found only in 5 hosts or less are grouped together.

### 2.3 Regression-ABC

This method follows that developed for application to phylodynamic datasets introduced in (Saulnier et al., 2017). In short, Approximate Bayesian Computation (ABC) is a likelihood-free method to infer parameter values from a given dataset (Beaumont, 2010). It consists in simulating many datasets, for which by definition the underlying parameters are known, and comparing them to the target dataset, the parameters of which we want to estimate. This comparison is often done by breaking the datasets into summary statistics. We use regression-ABC (Csilléry et al., 2012), which is divided into two steps. First, in the rejection step, only the simulated runs that are close enough from the target are kept. Second, a regression model is built on the remaining runs. Once we know how to map summary statistics to the parameter space, we can infer the parameters from any target dataset from which the same summary statistics can be extracts.

Using equation system (1), we ran numerical simulations to find the equilibrium prevalences of each of the 64 host classes (32 classes for each host type) for 50,001 parameter sets. We used large and uniform priors for the parameters (shown in Fig. S1). More specifically, we varied the competition intensity (our parameter of interest, *k* ∈ [−0.5, 0.5]) the transmission rate (*β* ∈ [0.5, 1.5]), the assortativity (*a* ∈ [0, 1]), the activity difference between host types (*h* ∈ [2, 20]) and the modifiers for the genotype-specific infection durations (*d*_*i*_ ∈ [0.6, 1]). The rate at which an infected host recovers from an infection by *i* is either *d*_*i*_ or *d*_*i*_(1 + *k*), if *i* is a LR-HPV in a coinfection with a HR-HPV. Since we assume an SIS model and estimate *β*, we renormalise our system by assuming that *d*_1_ = 1.

We compare three sets of summary statistics:

• the ranks set, which includes the 5 rank prevalences and the 5 total prevalence of each genotype, that is 10 summary statistics
• the comb set, which includes the rank set and the prevalences of the 32 combinations of genotypes, that is 42 summary statistics
• the all set, which includes the comb set for each of the two types of hosts, that is 84 summary statistics.

The first set is intended to mimic an approach that would ignore both the combinations of genotypes and the host groups (normal or superspreader). The second set is based on the type of data that could readily be accessed. The third is for a most optimistic scenario in which we would know which group each host belongs to. Importantly, we are using the same information used by earlier methods based on the prevalences of the genotype combinations.

Our regression-ABC algorithm, described in details in Saulnier et al. (2017), has two separate steps: first we reject a percentage of the simulated runs that are too far from the target, second we perform a regression on the remaining runs to obtain adjusted posterior distributions. We compared several levels of tolerance using a preliminary run of the model (with narrower priors) and identified 50% as an optimal cut-off for the rejection: lowering the tolerance did not improve the inference (measured via the fraction of runs where the target value ended up in the 95% Highest Posterior Density), whereas increasing it decreased the inference quality.

Still following our previous study (Saulnier et al., 2017), we then used a LASSO regression to adjust the posterior distribution. Although it performs a linear regression, it has the advantage to be less prone to over-fitting than more elaborate non-linear regressions, such as Support Vector Machines, neural networks or random forests. The LASSO adjustment was implemented using the glmnet R package and the ABC itself was performed using the abc package. In practice, one of the 50,001 runs was removed and used as a target, whereas the remaining runs were used to build the regression model (after performing a rejection step). We repeated the operation 100 times to generate 100 target datasets. For completeness, we also analysed 100 runs with only a single host type to compare our method to existing ones and investigate the robustness of the ABC to a mismatch between the model used to simulate the target model and the one used to build the regression model.

## 3 Results

### 3.1 Associations and competition intensity

We hypothesised that current methods, which implicitly assume a simple SI epidemiological model with cotransmission, may have difficulties to detect within-host competition between HPVs if there is another source of host heterogeneity than coinfection status. To test this hypothesis, we used our model to simulate target sets of genotype combination prevalences for known parameter values.

**Fig. 2:**
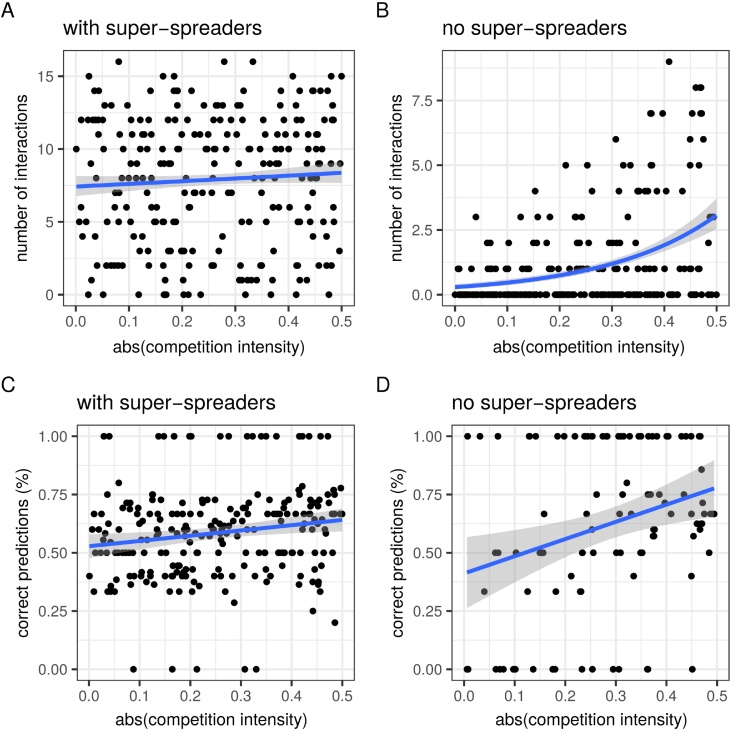
Total number of interactions detected with the association screening method (A and B) and fraction of these interactions that are consistent with model predictions (C and D). This analysis is ran for a model with two host types (A and C) or a single host type (B and D). The blue lines show the result of a linear model fit (A and B) and generalised linear model fit assuming a Poisson distribution of the outcome variable (C and D). Grey areas are prediction confidence intervals based on the standard error of the fit. These methods do not correct for multiple hypothesis testing, which could lower the number of interactions detected. In panels A and C, *h* = 1 and *a* = 0. We assume that there are *N* = 5000 hosts in the population.

Fig. 2 shows the performance of the association screening approach conceived by Vaumourin et al. (2014). With two host types, ‘normal-spreaders’ and ‘super-spreaders’, the number of significant interactions, i.e. the number of host types that show a prevalence that departs from the neutral expectation (*H*_0_), is independent from the intensity of the competitive interactions, |*k*| (Fig. 2A). Furthermore, the fraction of these predictions that correspond to what the analytical model would predict based on the nature of the interaction, i.e. the sign *k* (see Fig. S2), is always close to 50% (Fig. 2C). On the contrary, if we assume that there are no super-spreaders, then the number of significant interactions increases with competition intensity (Fig. 2B). The proportion of correct predictions also increases with competition intensity to reach a maximum estimated median of above 75% (Fig. 2D). This suggests that this method can be appropriate to detect strong competitive interactions in homogeneous host populations.

The Chi-square and GLM approaches are more qualitative: they either detect a difference with *H*_0_ or not. In Supplementary Fig. S5, we show that the GLM fails in both cases. For the chi-square approach, we do detect an increasing probability that the test is significant with increasing competition intensities (|*k*|) with a maximum of approximately 70%. As we will see later on, analysing the same target datasets with the ABC approach yields very different patterns.

### 3.2 Epidemiological model: single runs

We first show the prevalences of combination of genotypes in two scenarios: one with moderate interactions (parameter set #2 with the competition intensity parameter *k* ≈ 0.02, Fig. 1D) and another with strong interactions (parameter set #7 with *k* ≈ −0.41, Fig. 1E). When the interactions are weak, we clearly see the different ranks: uninfected hosts are on the top, then there is a row with the five singly infected host types, etc. When competition intensity increases, these ranks become impossible to distinguish. Fig. 1D also illustrates that each parasite genotype in this model has its own infection duration, since they do not all have the same prevalence in single infection (see rank 1 point data). We only show the total prevalence of each combination. However, for a given combination, the prevalence could be different in the two types of hosts (e.g. in the ‘super-spreader’ population, combinations of higher rank tend to be more prevalent).

Our goal is to infer the intensity and sign of the interaction between HR and LR genotypes (parameter *k*) in such a heterogeneous host population. To this end, we applied an ABC approach. As any Bayesian method, this means searching a prior distribution in the parameter space. This distribution is shown for all the key parameters in Fig. S1. We drew 50,001 parameter sets in this prior, used them to simulate equilibrium densities similar to the ones shown in Fig. 1D and E.

**Fig. 3:**
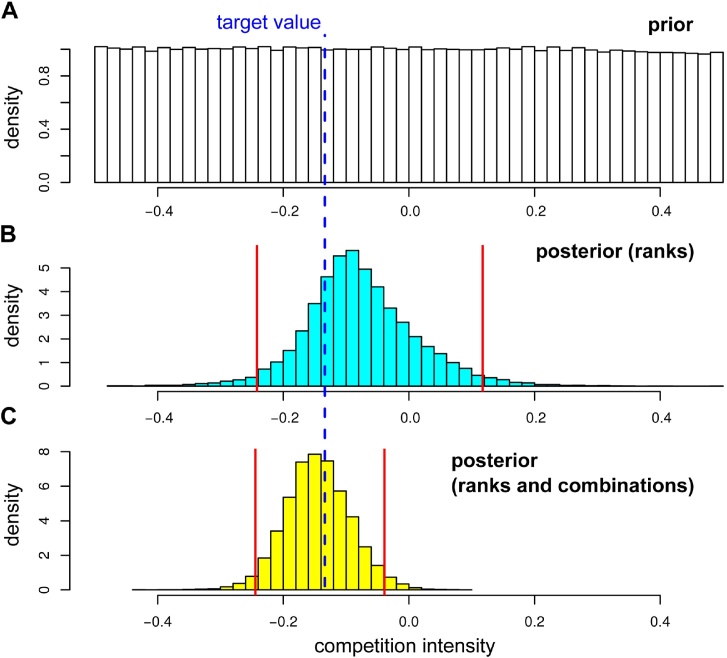
Inferring competition intensity (*k*). Prior (A) and posterior distributions using the ranks (B) or the comb set (C) of summary statistics. The dashed blue line shows the target value (*k* ≈ −0.13) and the red lines the 95% Highest Posterior Density (HPD).

Fig. 3 shows the results for parameter set #3 and illustrates how using more summary statistics helps to narrow the distribution from the prior for a dataset with 10,000 individuals. If we only use the ranks, we do narrow the prior distribution but its width remains large enough such that 0 (no interaction) cannot be ruled out from the 95% Highest Posterior Density (HPD), which can be seen as a credibility interval (Fig. 3B). Using the prevalence of the genotype combinations in addition to the prevalence of the infection ranks as summary statistics for the ABC allows us to narrow this interval and to exclude 0 from the 95% confidence interval (Fig. 3C). Using additional information, for example being able to distinguish between the two host types, would narrow it even more as we will see below.

### 3.3 Epidemiological model: cross-validation

The previous analysis was based on a single set of target parameters. Since all parameters may vary in a relatively large prior distribution (Fig. S1) and since *k* may be easier to infer in some settings, we assessed the performance of the ABC approach following a leave-one-out cross-validation procedure, where we treated one simulation as observed data and the remaining as learning data. We varied the number of sampled individuals and used 100 targets for each. Furthermore, we analyse a third set of summary statistics involving the prevalences of infection ranks and genotype combinations for the two hosts sub-populations (see the Methods).

**Fig. 4:**
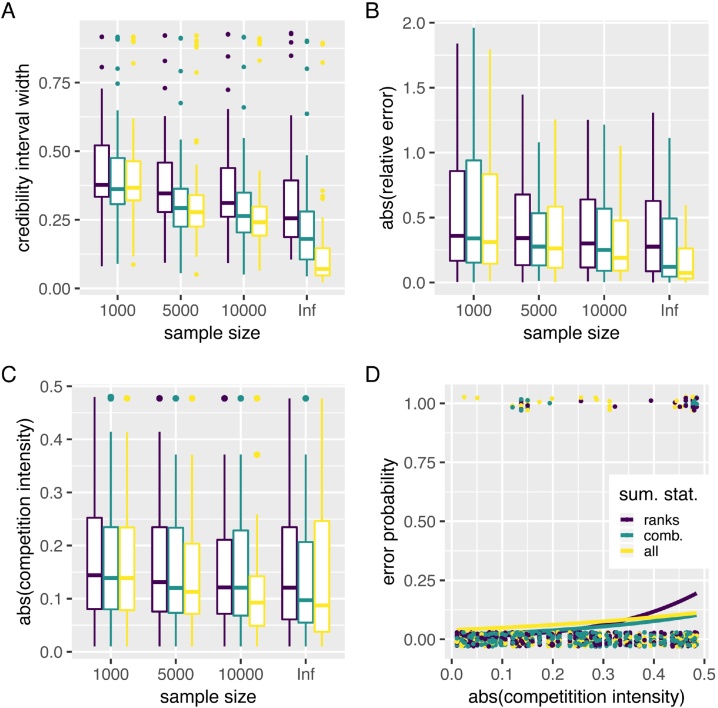
ABC inference precision over 100 runs. (A) 95% Highest Posterior Density (HPD), (B) absolute value of the relative error, (C) average of the absolute value of competition intensity in runs where 0 is in the 95% HPD and (D) runs for which the target value lies outside the 95% HPD. Colours indicate the summary statistics used for the ABC. In D, the lines show the results of generalised linear models assuming a binomial distribution of the outcome variable.

As expected, the width of the 95% HPD for the estimate of competition intensity decreased with the number of host sampled (Fig. 4A). On the same figure, we see that including more summary statistics also decreased the width of this interval, especially when the exact prevalence is known (infinite population size assumption).

In terms of the relative error made when estimating the competition intensity parameter (*k*), we found a similar effect with a lower error when more hosts were sampled or more summary statistics were involved (Fig. 4B). This effect is the clearest when using all the summary statistics and the exact prevalences (the ‘inf’ population size). In general, we see that increasing the number of summary statistics does not help when few hosts are sampled (all three sets are similar when *N* = 1000) and that using the prevalences of the genotype combinations only improves the analysis if enough hosts are sampled (5000 or more). The relative error also tended to decrease with competition intensity.

If we focus on the runs for which we could not exclude an absence of interaction (i.e. *k* = 0 lied within the 95% HPD), we see that the number of such runs decreased as the number of summary statistics increased (Fig. S3). We also see that, in these runs, competition intensity decreased with the sample size and with the number of summary statistics involved (Fig. 4C). Notice that for large sample sizes, 95% HPD are narrower, which makes it more difficult to exclude an absence of competitive interactions.

Finally, the probability to make an error in the inference, which we define as having the target value outside the 95% HPD, was close to the expected 5% (6.25% with the ranks and 5% with the comb sets). This probability slightly increased with competition intensity, especially when the genotype combination prevalences were ignored in the ABC (Fig. 4D). Therefore, we have the somewhat unexpected result that genotype combination data is more important to analyse datasets where competitive interactions are particularly strong. This could be due to the fact that extreme scenarios with parameter values at the edge of the prior are more difficult to infer because there is less data to train the regression model.

### 3.4 Removing host heterogeneity

We next used the ABC approach to reanalyse the target sets with a single host type shown in Fig. 2B. This allowed us to do more than simply compare methods. Indeed, in our prior for the ABC, the heterogeneity parameter is greater than 2. This means there is a mismatch between the model we assumed for the ABC (2 host types with some heterogeneity between them) and that used to generate the target data (1 host type). We can therefore evaluate the robustness of the inference method to a small error in model specification.

**Fig. 5:**
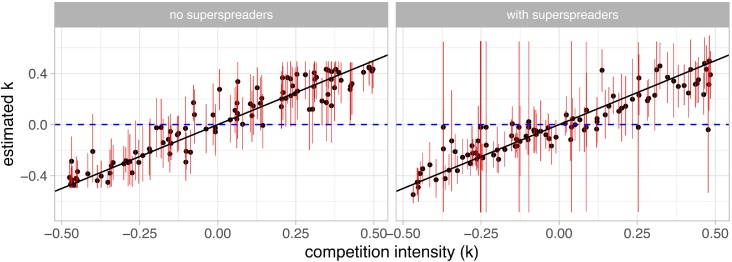
Inferring competition parameter (*k*) in, a setting with (A) and without (B) host behavioural heterogeneity. Red lines show the 95% credibility interval and the blue line shows the absence of interaction (*k* = 0). The target runs are identical to those in Fig. 4, Fig. 2 with *N* = 5000 hosts and the comb set of summary statistics.

We investigated the relationship between genotype competition intensity (*k*) and our ability to reject an absence of interaction (*k* = 0) from the 95% HPD in a situation with two host types and one host type in the target dataset. Priors were identical to the other analyses and shown in Fig. S1. In both situations, cases where the true competition value was not in the 95% HPD interval were close to 5% as in the other runs. We then investigated how often an absence of competition (that is *k* = 0) could be rejected. This is similar to the *H*_0_ tested by Vaumourin et al. (2014). We found that we could detect competition for 55% of the target values in a model with super-spreaders and for 63% of the target values in model with only a single host type. In the latter we also made one error, i.e. inferred a positive interaction for a negative target. This is because in this specific parameter set, the modifiers for the infection duration of the two LR genotypes (*d*_2_ and *d*_5_) were low, whereas that of the HR were all high, therefore perfectly mimicking a competition interaction. Fig. 5 also shows that, as expected, the ability to reject *H*_0_ increased with competition intensity. Overall, removing the heterogeneity in the data due to differences in host behaviour does increase our ability to detect competitive interactions.

## 4 Discussion

Multiple infections are known to affect the virulence of an infection (Griffiths et al., 2011), the spread of infectious diseases (Abu-Raddad et al., 2006) and their evolution (Alizon et al., 2013). This is due to the fact that when sharing a host, parasites can interact in various ways such as competing for host resources, exploiting molecules they produce or even indirectly via cross-reactive immune response (Mideo, 2009). The goal of this study was to determine to what extent the prevalence of specific genotype combinations can inform us on the net effect of all these interactions.

By generating prevalence data from a mechanistic epidemiological model, we were able to first test the power of existing heuristic methods based on neutral distributions that implicitly assume a Susceptible-Infected (SI) model with co-transmission and only a single type of hosts. We showed that introducing host heterogeneity into the model can modify the distribution of genotype combination prevalences in a way that makes within-host interactions between genotypes largely undetectable. This therefore corroborates a limitation often mentioned in such studies, as departures from ‘neutral’ distributions (*H*_0_) may not necessarily be due to interaction between parasite genotypes.

We then used an ABC approach to infer parameters from the model. We showed that this yields more consistent results than existing methods. As expected, the accuracy of the method increases with the number of hosts sampled. We also showed that using the prevalence of all the combinations of host classes tends to decrease the error made compared to using only the prevalence of infection ranks. Finally, adding information in the target data about host type (‘super-spreader’ or ‘normal-spreader’) can further improve the power of the inference.

The fact that decent results can be obtained by only using infection ranks may seem surprising considering the difficulty from existing models to infer interactions. This could mean that accounting for host behavioural heterogeneity is more important than adding additional information via the genotype combinations. Another reason could be that we here use the same model to generate the target dataset and the learning datasets, which facilitates the ABC inference. However, we do show that our inference method performs very well to infer competitive interactions when there is a slight mismatch between the true model and that used in the ABC. Finally, this could also mean that there is room for inference improvement in our choice of summary statistics. Indeed, as shown in Saulnier et al. (2017), designing specific summary statistics can help improve the inference of a given parameter.

As illustrated by Fig. S4, our ability to extract information from the data varied widely across parameters. For the interaction parameter (*k*), the inference reduced the initial 95% HPD of the prior by 66%. In comparison, this was less than for the transmission probability (*β*, 75%), but much better than for the assortativity parameter (*a*, 45%), host heterogeneity (*h*, 38%) or the individual recovery rates of each genotype *i* (*d*_*i*_, 13%).

There are several ways to extend this framework. One would be to use more powerful non-linear machine learning regression techniques, such as neural networks. However, these may be more difficult to parameterise than the linear one we used here. Furthermore, even though it contains several parameters, our model remains relatively simple compared to the power of these algorithms.

Here, we have also generally assumed that the epidemiological model is known. There are two ways to extend this. One can be to perform rigorous model comparison to see whether a simpler model (for instance with a single host type), might not fit the data better. This could be done readily using regression-ABC, for instance with random forests (Pudlo et al., 2016). Another extension would be to use an agent-based model with sophisticated agent behaviours to generate a richer dataset. This would be useful in itself to generate test runs with known parameter values to further test the power of our method on more noisy data. It would also allow to control for biases related to the contact network structure between hosts and the dynamical aspect of sexual partnerships that have been shown to interfere with the detection of coinfection interactions (Malagón et al., 2016).

Finally, the next step is, of course, to test this model using actual epidemiological data. Even in the case of HPV, analysing real data will require to add several processes we chose to ignore here. First, HPV detection tests may exhibit cross-reactivity between HPV types, thus inflating the prevalence of some genotype combinations. This effect if well described and can be handled for each detection test (Eklund et al., 2014). Second, when hosts are infected by many HPV types, some of these may not be detected, thus decreasing the prevalence of high-rank infections. This effect is more subtle and would require to be inferred in the model. Finally, one of the advantages of the ABC is that it can accommodate different types of dataset. In the case of coinfections, on possibility could be to include information about virus loads (Xi et al., 2009), which would also help explore the within-host compartment (Sofonea et al., 2015). Furthermore, allowing for longitudinal follow-ups would also open the door to many more summary statistics (Man et al., 2019). In general, these studies could have a strong impact due to the debate on potential for HPV type replacement following mass vaccination (Murall et al., 2015, Tota et al., 2016, Man et al., 2019)

We mainly referred to HPV but other systems could be studied, in particular coinfections between different parasite species and wild host species such as mice (Knowles et al., 2013, Råberg et al., 2017) or sheep (Hayward et al., 2014). However, it is important to stress that the underlying epidemiological model must be consistent with the life-history of the parasite(s). Indeed, these could generate sources of heterogeneity similar to the one we introduced via host behaviour.

Overall, ABC and machine learning allow us to extract the information from the equilibrium prevalence values of all genotype combinations. Therefore, combining coinfection modelling with epidemiological data can bring new elements to the controversy regarding the importance of interactions between HPV types.

## Supporting information

In addition to the supplementary figures, the archive SupplementaryMaterials.zip contains all the scripts used to generate the cross-validation results and plot the figure, along with a master table containing the results for each scenario (summary statistics used, tolerance, parameter ranges).

## Funding

This project has received funding from the European Research Council (ERC) under the European Union's Horizon 2020 research and innovation program (grant agreement No 648963) with additional funding from the CNRS and the IRD.

## Conflict of interest disclosure

The authors of this preprint declare that they have no financial conflict of interest with the content of this article. SA and CLM are recommenders for PCI Evol Biol and PCI Ecology.
