# Supplementary material for: Detecting within-host interactions from genotype combination prevalence data
Source: Epidemics. 2019 Dec;29:100349. doi: 10.1016/j.epidem.2019.100349 (PMC6899502; doi:10.1016/j.epidem.2019.100349)
Supplement: Supplementary file 1 [file mmc1.pdf]

Supplementary text to *Detecting within-host interactions using genotype combination prevalence data*

Contents

A Supplementary Information **1**
A.1 HPV coinfections and interactions 1
A.2 Deriving the master equation for epidemiological dynamics . 2
A.2.1 Infection rates 3
A.2.2 Recovery rates 4

B Supplementary Figures **8**

A Supplementary Information

A.1 HPV coinfections and interactions

Although our approach can be applied to many systems, we focus here on genital infections caused by different types of human papillomaviruses (HPVs) for several reasons. First, multiple infections between HPV types are common (Fig 1A) and well described thanks to screening for HPV-induced cancers (Vaccarella et al., 2010, Chaturvedi et al., 2011, Dickson et al., 2013). Second, their prevalences are relatively stable through time (Alemany et al., 2014). Third, HPV evolutionary rates are generally slow, which limits within-host evolution and facilitates detection (Bravo et al., 2010). Fourth, the existence of within-host interactions between HPV types is strongly debated, especially in the context of vaccination, given that they may affect a potential parasite evolutionary response (Murall et al., 2015).

Because of the high prevalence of coinfections and, more generally, because of the low immunogenicity and low pathogenesis of acute HPV infections (Alizon et al., 2017), some believe HPV between-types interactions in coinfected hosts to be negligible, which seems consistent

with some epidemiological data (Chaturvedi et al., 2011). However, pre-vaccine and vaccine studies have shown that there is limited natural cross-reactivity between phylogenetically related HPV types and that vaccines confer partial cross-immunity against non-target types (Herrero, 2009, Wheeler et al., 2012, Beachler et al., 2016). This means that there could be apparent competition mediated by the immune system. At the cellular level, recent data supports the existence of superinfection, that is one HPV type excluding the other from the cell (Biryukov & Meyers, 2018). For some types, virus loads also seem to differ in single and in coinfections (Xi et al., 2009), which could impact the host transmission and recovery rates. There is also indirect epidemiological evidence. First, infection by HPV is known to affect the risk of contracting other infections (Rousseau et al., 2001, Méndez et al., 2005, Tota et al., 2016) and to decrease the recovery rate of another type in coinfection (Trottier et al., 2008). Second, HPV coinfections may interfere with chronic infection and cancer. For example, when oncogenic 'high-risk' (HR) HPV types coinfect with non-oncogenic 'low-risk' (LR) types, time to diagnosis is longer and the risk of progression to cancer is lower (Sundström et al., 2015).

In summary, there are reasons to hypothesise that HPV types might interact when coinfecting a host and that these interactions could be large enough to affect the prevalence of some genotype combinations. Detecting or ruling out such interactions would also have a strong impact in the field, especially in the context of vaccination against specific HPV types as they could mean a risk for type replacement. Importantly, our approach has no explicit within-host component and is therefore unable to detect a specific interaction. Instead, what it can detect is the overall effect of all the potential within-host interactions between genotypes.

A.2 Deriving the master equation for epidemiological dynamics

We here explicit the notations used in the main text. This is directly based on earlier work and explained in further details in (Sofonea et al., 2015).

The master equation of between-host dynamics introduced in the main text is:

$$\frac{\mathrm{d}}{\mathrm{d}t}\mathbf{y} = \mathbf{\Phi}.\left(\mathbf{y} \otimes \mathbf{y}\right) - \left(\mathbf{\Psi}.\mathbf{y}\right) \odot \mathbf{y} + \left(\mathbf{\Xi} - \mathbf{\Theta}\right).\mathbf{y}. \tag{S1}$$

The expression of each matrix are the following:

$$\begin{cases} \mathbf{\Phi} \coloneqq \left(\beta_{\mathfrak{r}(j),\mathfrak{d}(j),i}\right)_{(i,j)\in[\![0;2^{2n}-1]\!][\![0;2^{n}-1]\!]}, \\ \mathfrak{r}(j) \coloneqq \left\lfloor \frac{j}{2^n} \right\rfloor, \quad \mathfrak{d}(j) \coloneqq \mathrm{mod}_{2^n}(j), \\ \mathbf{\Psi} \coloneqq \left(\sum_{\ell=0}^{2^n-1} \beta_{i,j,\ell}\right)_{(i,j)\in[\![0;2^n-1]\!]^2}, \\ \mathbf{\Xi} \coloneqq \left(\theta_{i,j}\right)_{(i,j)\in[\![0;2^n-1]\!]^2}, \\ \mathbf{\Theta} \coloneqq \left(\delta_{i,j}\sum_{\ell=0}^{2^n-1}\theta_{i,\ell}\right)_{(i,j)\in[\![0;2^n-1]\!]^2}. \end{cases} \tag{S2}$$

Here, $\beta_{r,d,i}$ is an infection rate. It indicate the rate at which a ‘donor’ host infected by a combination of genotypes d can create an infected host of combination of genotypes i by infecting a ‘receiver’ host infected by a combination of gentoypes r.

$\theta_{d,i}$ is a clearance rate, which captures a flow from hosts infected by a combination of genotypes d towards another class of hosts infected by a combination of genotypes i.

$\delta_{a,b}$ refers to the Kronecker's delta, which is 1 if $a = b$ and 0 otherwise.

$\mathfrak{r}(j)$ and $\mathfrak{d}(j)$ are operators created to transform a set of genotypes into an integer through a binary code. Indeed, for n different genotypes, there exist exactly 2^n different host (and inoculum) classes. We use the property that natural number can be written using the binary numeral system. For further details, see (Sofonea et al., 2015).

Note that the $\mathbf{\Phi}$ matrix has a nested structure (donor host classes are nested into receiver host classes) that requires arithmetical calculation on indices, whence the $\mathfrak{r}$ and $\mathfrak{d}$ functions.

To further understand these notations, we need to make explicit the infection rates and recovery rates, as originally developed in (Sofonea et al., 2015).

A.2.1 Infection rates

Using our binary labelling (Sofonea et al., 2015), the labelled form of the infection rates is the following

$$\beta_{r,d,i} = \beta n_d^{-n} \sum_{p=0}^{2^n-1} \min_{k\in[\![1,n]\!]} \left(\delta_{c_{d,k},c_{d,k}+c_{p,k}-c_{d,k}c_{p,k}}\right) \delta_{i,\phi(r,p)} n_p \times \prod_{k=1}^{n} \left(2c_{p,k} - 1 + (1-c_{p,k})\, n_d\right)$$

where

1. β is the constant transmission factor,

2. n_j is the rank of the inoculum or host class j,

3. n is the number of genotypes,

4. $\sum_{p=0}^{2^n-1}$ is the sum over all inocula,

5. $\min_{k \in [\![1;n]\!]} \left(\delta_{c_{d,k}, c_{d,k}+c_{p,k}-c_{d,k}c_{p,k}}\right)$ cancels out whenever a genotype belongs to p but not to d (ensuring that inoculum p can be produced by donor host d),

6. $\delta_{i,\phi(r,p)}$ cancels out whenever host class r does not turn into host class i when infected by inoculum class p,

7. $\prod_{k=1}^{n}$ is the product over all genotypes (nested in the inocula),

8. $(2c_{p,k} - 1 + (1 - c_{p,k})\, n_d)$ is the product over all genotypes of d depending on the presence or absence in p.

A.2.2 Recovery rates

Contrarily to the transmission process, the recovery events occur at different rates depending on the genotype involved (see the main text). Assuming that genotypes can only be cleared one at a time, the labelled form of the recovery rates is the following

$$\theta_{d,i} := (1 - \delta_{d,i}) \sum_{j=1}^{n} d_j \kappa_{d,j} \delta_{i,\phi\left(0, c_{d,j}(d-2^{j-1})\right)},$$

where

1. $(1 - \delta_{r,i})$ cancels out if the recovery event is trivial (the recovering class is already the output),

2. $\sum_{j=1}^{n}$ is the sum over all genotypes,

3. d_j is the recovery rate of genotype j,

4. $\kappa_{d,j}$ is the modifier on d_j depending on the other genotypes present in class d (in this study, it is always 1 if j is a HR genotype, and it can be equal to k_j if j is a LR genotype and there is a HR genotype in class d),

5. $\delta_{i,\phi\left(0, c_{d,j}(d-2^{j-1})\right)}$ cancels out whenever host class d does not turn into host class i when losing genotype j.

References

Alemany, L., de Sanjosé, S., Tous, S., Quint, W., Vallejos, C., Shin, H.-R., Bravo, L. E., Alonso, P., Lima, M. A., Guimerà, N., Klaustermeier, J., Llombart-Bosch, A., Kasamatsu, E., Tatti, S. A., Felix, A., Molina, C., Velasco, J., Lloveras, B., Clavero, O., Lerma, E., Laco, J., Bravo, I. G., Guarch, R., Pelayo, A., Ordi, J., Andújar, M., Sanchez, G. I., Castellsagué, X., Muñoz, N., Bosch, F. X., & on Behalf of the RIS HPV TT Study Group (2014). Time trends of human papillomavirus types in invasive cervical cancer, from 1940 to 2007. *Int J Cancer*, *135*, 88–95. doi:10.1002/ijc.28636.

Alizon, S., Murall, C. L., & Bravo, I. G. (2017). Why Human Papillomavirus Acute Infections Matter. *Viruses*, *9*, 293. doi:10.3390/v9100293.

Beachler, D. C., Jenkins, G., Safaeian, M., Kreimer, A. R., & Wentzensen, N. (2016). Natural acquired immunity against subsequent genital human papillomavirus infection: A systematic review and meta-analysis. *J Infect Dis*, *213*, 1444–1454. doi:10.1093/infdis/jiv753.

Biryukov, J., & Meyers, C. (2018). Superinfection Exclusion between Two High-Risk Human Papillomavirus (HPV) Types During a Co-Infection. *J Virol*, (p. in press). doi:10.1128/JVI.01993-17.

Bravo, I. G., de Sanjosé, S., & Gottschling, M. (2010). The clinical importance of understanding the evolution of papillomaviruses. *Trends Microbiol*, *18*, 432–8. doi:10.1016/j.tim.2010.07.008.

Chaturvedi, A. K., Katki, H. A., Hildesheim, A., Rodríguez, A. C., Quint, W., Schiffman, M., Doorn, L.-J. V., Carolina Porras, . S. W., Gonzalez, P., Sherman, M. E., Herrero, R., & the CVT Group (2011). Human papillomavirus infection with multiple types: Pattern of coinfection and risk of cervical disease. *J Infect Dis*, *203*, 910–920. doi:10.1093/infdis/jiq139.

Dickson, E. L., Vogel, R. I., Bliss, R. L., & Downs, L. S. (2013). Multiple-type Human Papillomavirus (HPV) infections: A cross-sectional analysis of the prevalence of specific types in 309,000 women referred for HPV testing at the time of cervical cytology. *Int J Gynecol Cancer*, *23*, 1295–1302. doi:10.1097/IGC.0b013e31829e9fb4.

Herrero, R. (2009). Human papillomavirus (HPV) vaccines: limited cross-protection against additional HPV types. *J. Infect. Dis.*, *199*, 919–922. doi:10.1086/597308.

Méndez, F., Muñoz, N., Posso, H., Molano, M., Moreno, V., van den Brule, A. J. C., Ronderos, M., & Meijer, C. (2005). Cervical coinfection with Human Papillomavirus (HPV) types and possible implications for the prevention of cervical cancer by HPV vaccines:. *J Infect Dis*, *192*, 1158–1165. doi:10.1086/444391.

Murall, C. L., Bauch, C. T., & Day, T. (2015). Could the human papillomavirus vaccines drive virulence evolution? *Proc Biol Sci*, *282*, 20141069. doi:10.1098/rspb.2014.1069.

Rousseau, M.-C., Pereira, J. S., Prado, J. C. M., Villa, L. L., Rohan, T. E., & Franco, E. L. (2001). Cervical coinfection with Human Papillomavirus (HPV) types as a predictor of acquisition and persistence of HPV infection. *J Infect Dis*, *184*, 1508–1517. doi:10.1086/324579.

Sofonea, M., Alizon, S., & Michalakis, Y. (2015). From within-host interactions to epidemiological competition: a general model for multiple infections. *Philos Trans R Soc Lond B*, *370*, 20140303. doi:10.1098/rstb.2014.0303.

Sundström, K., Ploner, A., Arnheim-Dahlström, L., Eloranta, S., Palmgren, J., Adami, H.-O., Ylitalo Helm, N., Sparén, P., & Dillner, J. (2015). Interactions between high- and low-risk HPV types reduce the risk of squamous cervical cancer. *J Natl Cancer Inst*, *107*, djv185. doi:10.1093/jnci/djv185.

Tota, J. E., Ramanakumar, A. V., Villa, L. L., Richardson, H., Burchell, A. N., Coutlée, F., & Franco, E. L. (2016). Cervical Infection With Vaccine-Associated Human Papillomavirus (HPV) Genotypes as a Predictor of Acquisition and Clearance of Other HPV Infections. *J Infect Dis*, . doi:10.1093/infdis/jiw215.

Trottier, H., Mahmud, S., Prado, J. C. M., Sobrinho, J. S., Costa, M. C., Rohan, T. E., Villa, L. L., & Franco, E. L. (2008). Type-Specific Duration of Human Papillomavirus Infection: Implications for Human Papillomavirus Screening and Vaccination. *J Infect Dis*, *197*, 1436–1447. doi:10.1086/587698.

Vaccarella, S., Franceschi, S., Snijders, P. J. F., Herrero, R., Meijer, C. J. L. M., Plummer, M., & Group, t. I. H. P. S. S. (2010). Concurrent infection with multiple Human Papillomavirus types: Pooled analysis of the IARC HPV prevalence surveys. *Cancer Epidemiol Biomarkers Prev*, *19*, 503–510. doi:10.1158/1055-9965.EPI-09-0983.

Wheeler, C. M., Castellsagué, X., Garland, S. M., Szarewski, A., Paavonen, J., Naud, P., Salmerón, J., Chow, S.-N., Apter, D., Kitchener, H.,

Teixeira, J. C., Skinner, S. R., Jaisamrarn, U., Limson, G., Romanowski, B., Aoki, F. Y., Schwarz, T. F., Poppe, W. A. J., Bosch, F. X., Harper, D. M., Huh, W., Hardt, K., Zahaf, T., Descamps, D., Struyf, F., Dubin, G., & Lehtinen, M. (2012). Cross-protective efficacy of HPV-16/18 AS04-adjuvanted vaccine against cervical infection and precancer caused by non-vaccine oncogenic HPV types: 4-year end-of-study analysis of the randomised, double-blind PATRICIA trial. *Lancet Oncol*, *13*, 100–110. doi:10.1016/S1470-2045(11)70287-X.

Xi, L. F., Edelstein, Z. R., Meyers, C., Ho, J., Cherne, S. L., & Schiffman, M. (2009). Human Papillomavirus types 16 and 18 DNA load in relation to coexistence of other types, particularly those in the same species. *Cancer Epidemiol Biomarkers Prev*, *18*, 2507–2512. doi:10.1158/1055-9965.EPI-09-0482.

B Supplementary Figures

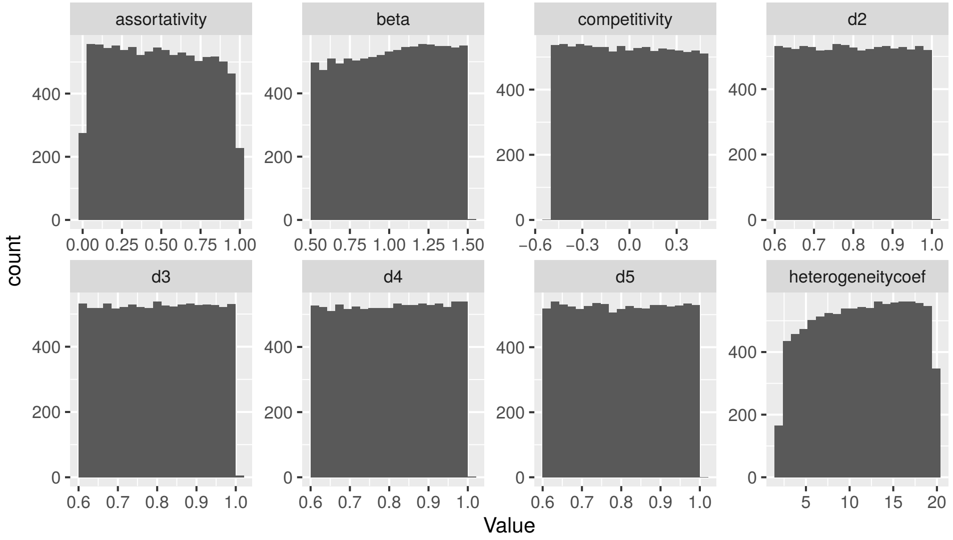

Fig S1. Prior distributions for all the parameters. The same priors are used to generate target datasets and training datasets.

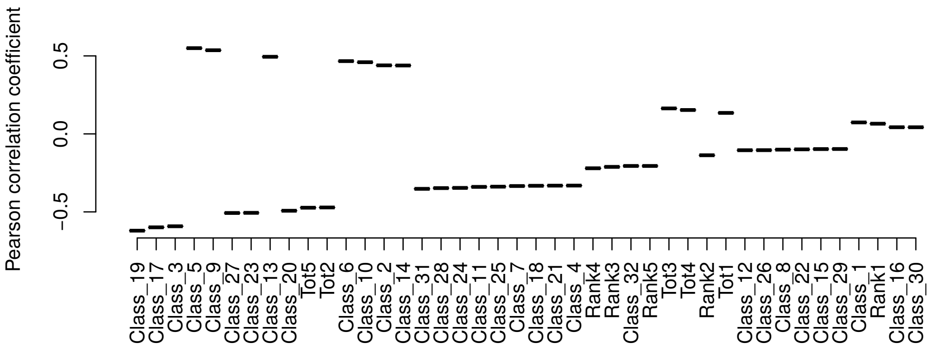

Fig S2. Correlation between competition intensity and (combination, rank or genotype) prevalence. Values show the Pearson correlation coefficient obtained using 1,000 parameter sets from the ABC training dataset (priors in Figure S2).

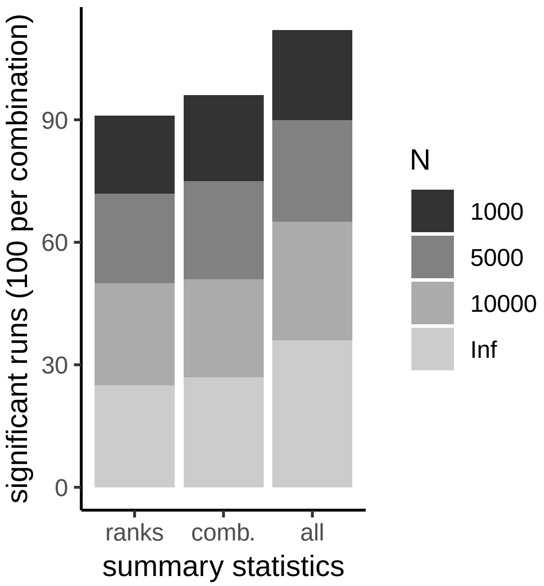

Fig S3. Number of runs where 0 can be excluded from the 95% HPD. Increasing the sample size and the number of summary statistics increases the number of such ‘significant’ runs. For each combination of N and summary statistics, 100 runs were performed.

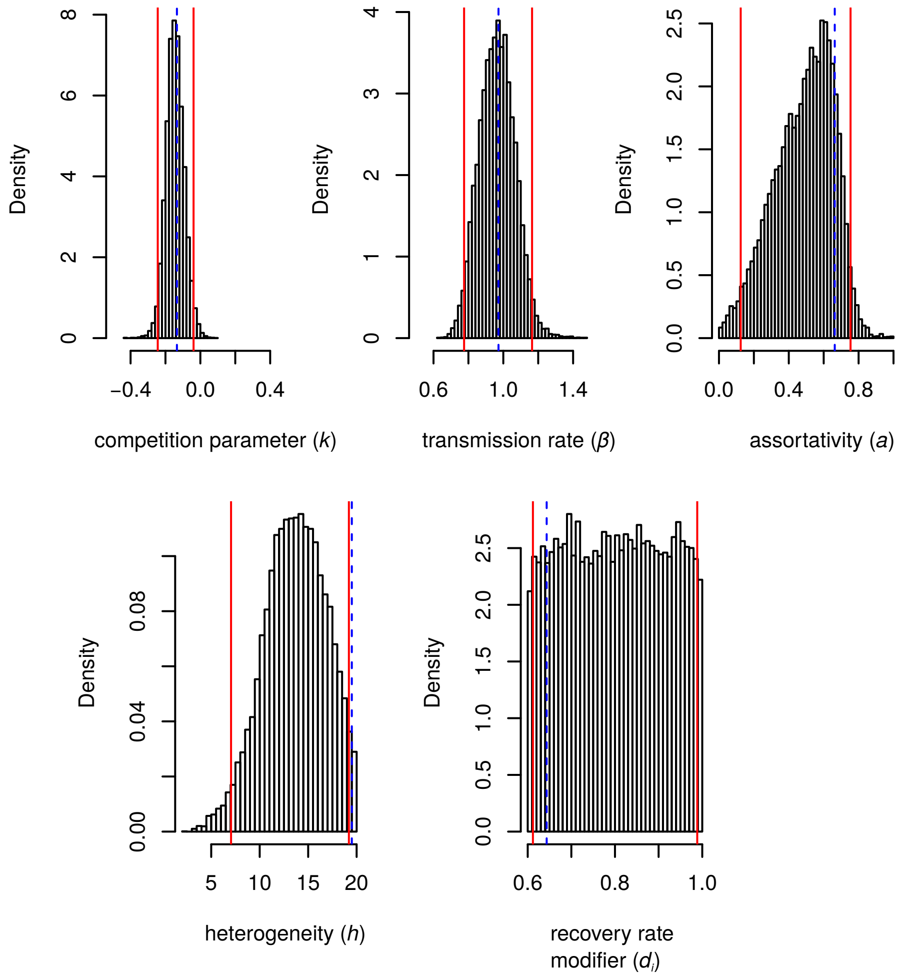

Fig S4. Inferring model parameters using the comb summary statistics. We use parameter set #3 as the target and the remaining 50,000 sets to perform the ABC. The dashed blue lines show the target values and the red lines show the 95% Highest Posterior Density (HPD).

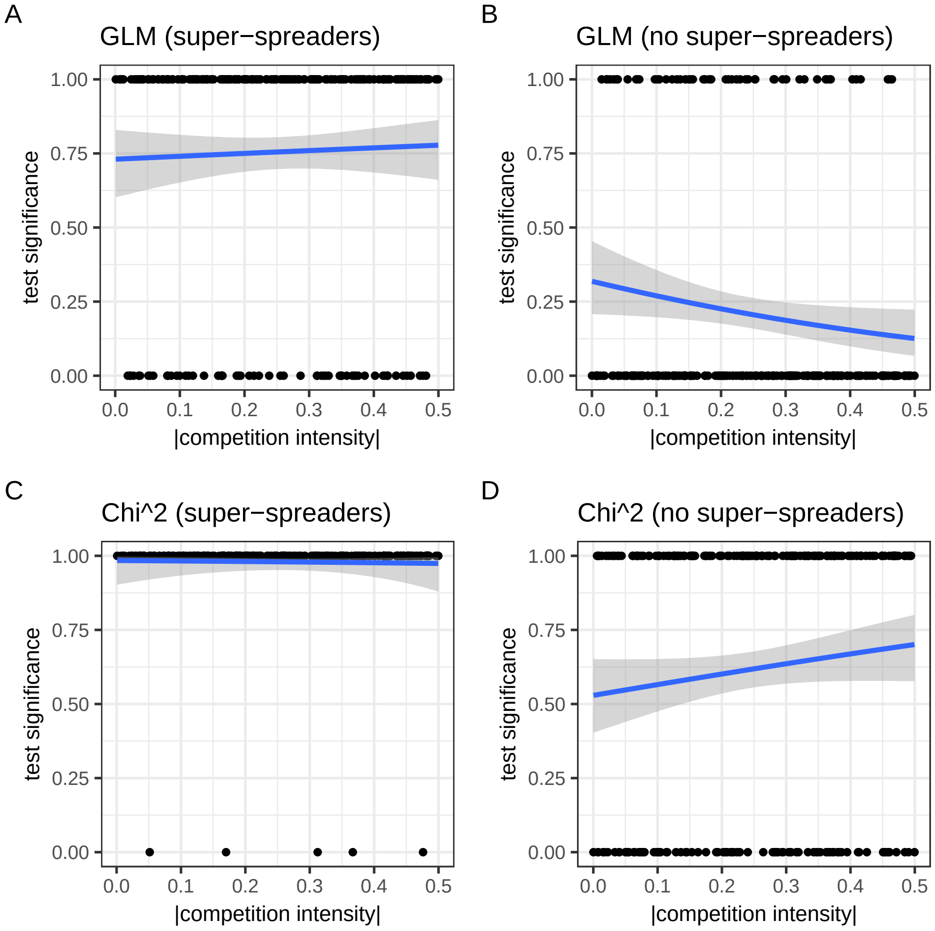

Fig S5. Significancy of the GLM (A and B) and the chi-square (C and D) approaches. This analysis is run for a model with two host types (A and C) or a single host type (B and D). In panels A and C, $h = 1$ and $a = 0$.
